# Assessment of liver stiffness in patients with HCV and mixed cryoglobulinemia undergoing rituximab treatment

**DOI:** 10.1186/1479-5876-12-21

**Published:** 2014-01-24

**Authors:** Cristina Stasi, Elisa Triboli, Umberto Arena, Teresa Urraro, Antonio Petrarca, Laura Gragnani, Giacomo Laffi, Anna Linda Zignego

**Affiliations:** 1 Interdepartmental Center for Systemic Manifestations of Hepatitis Viruses MASVE, University of Florence, Florence, Italy; 2 Department of Experimental and Clinical Medicine, University of Florence, Largo Brambilla 3, 50134 Florence, Italy

**Keywords:** Hepatitis C virus infection, Elastography, Rituximab, Mixed cryoglobulinemia

## Abstract

**Introduction:**

Mixed cryoglobulinemia (MC) is a HCV-related lymphoproliferative disorder generally associated with advanced liver disease. Liver stiffness has been significantly correlated with histopathological stage of fibrosis. Moreover, it was influenced by necroinflammatory activity. Rituximab (RTX) is a chimeric anti**-**CD20 monoclonal antibody inducing transient B lymphocytes depletion that was shown to be useful and safe in the majority of HCV MC patients, leading also to improvement of cirrhotic syndrome. Aim of this study was to evaluate the modifications of liver stiffness following RTX treatment in HCV-related MC patients.

**Materials and methods:**

Fourteen consecutive patients (10 F, 4 M; mean age 60.43 ± 43) with HCV-related chronic hepatitis (n = 10) or cirrhosis (n = 4) and MC, eligible for RTX treatment, were prospectively enrolled. Intravenous injection of 1 g of RTX was performed at day 0 and at day 15. Assessment of stiffness was carried out by Fibroscan^®^ (Echosens, Paris-France) at baseline, 15 days after the first infusion, and at month 1, 3 and 6 after therapy.

**Results:**

MC symptoms significantly improved during the study, especially during the first 3 months. Liver stiffness observed 3 months after treatment was significantly reduced when compared with pre-treatment values (p = 0.01). This difference disappeared after 6 months of follow-up. Cytofluorimetric analysis showed a decrease of CD19+ peripheral blood cells, with the nadir at month 3 after therapy and B cell compartment reconstitution after 6 months.

**Conclusion:**

This study, for the first time showed that RTX-treatment in HCV-related MC induces a reduction of liver stiffness that is strictly associated with the B-cell depletion.

## Introduction

It is known that Hepatitis C virus (HCV) can infect mononuclear cells [1-3] and is associated with several autoimmune/lymphoproliferative disorders, whose prototype is mixed cryoglobulinemia (MC) [4]. MC is a both autoimmune and B-cell lymphoproliferative disorder characterized by immune complexes that reversibly precipitate at low temperature (cryoglobulins). A striking association between HCV and MC was shown [5,6]: HCV infection was detected in a percentage from 70% to >90% of MC patients and cryoglobulins in about half of HCV patients [7,8]. MC is a benign, but pre-lymphomatous condition whose clinical manifestations - the so-called mixed cryoglobulinemia syndrome, observed in a minority of total MC cases - are secondary to a systemic vasculitis of the small/medium size vessels [9]. It has been shown that MC can evolve to non-Hodgkin lymphoma (NHL) in 5-10% of patients without anti-HCV terapy [9] and that the overall risk of NHL in patients with MC is about 35 times higher than in the general population [10]. Among the most common symptoms, there are fatigue, arthralgias and purpura (Meltzer’s triad). Most severe cases are associated with impairment of the kidney and central and/or peripheral nerves [8,11]. Anti-HCV therapy with pegylated interferon (Peg-IFN) and ribavirin (RBV) is considered the first therapeutic option in the treatment of mild to moderate HCV-related MC syndrome [12].

When therapy is contraindicated or not tolerated (i.e, for the severity of disease, advanced age, general clinical conditions), other therapeutic options are chosen, including, for first, immunosuppressant and antiflogistic drugs [12]. During the last decade, increasing importance was attributed to the use of the B-cell specific immunosuppressant Rituximab (RTX) [13]. RTX is a chimeric monoclonal antibody that binds to CD20 antigen on the B cell surface determining their depletion, with reconstitution generally starting from the sixth month [14]. Petrarca *et al*. [15] previously showed that therapy with RTX is not burdened by significant liver toxicity also when administered to patients with advanced liver disease. More interestingly, it was shown that in patients with HCV MC and clinically evident cirrhosis, RTX was able to induce improvement of cirrhosis syndrome, with Child-Pugh score reduction [15]. The reasons for such an observation are at present unknown. Transient elastography (TE) has been proposed as a possible tool for diagnosis and monitoring of non-significant fibrosis or severe fibrosis/cirrhosis in patients with chronic HCV infection [16-18]. Liver stiffness values have been significantly correlated with histopathological staging of liver biopsy, with excellent diagnostic accuracy in distinguishing absent/mild and advanced fibrosis [19,20], though Arena et al. showed that liver stiffness was influenced by necro-inflammatory activity [16].

Aim of this study was to evaluate the change of liver stiffness values during and after treatment with RTX in patients with HCV MC.

## Materials and methods

Fourteen consecutive patients with chronic liver disease and HCV type II MC (10 women and 4 men) for whom the antiviral treatment was contraindicated or not tolerated, were consecutively enrolled at the MASVE Center outpatient clinic, University of Florence, Florence, Italy.

MC syndrome was diagnosed according to previously described criteria [15,21]. Serum cryoglobulin levels and characterization, levels of complement fractions, rheumatoid factor, and autoantibodies were evaluated as described [22-24]. The mean duration of MC was 40.14 months (range, 7–174 months). The most relevant clinical manifestations are listed in Table 1.

**Table 1:** Principal mixed cryoglobulinemia manifestations (clinical and biohumoral) diagnosed before treatment with rituximab and 3 months after therapy

**MC manifestations**	**Pre-treatment**	**3 months**
	**(%)**	**(%)**
***Clinical***		
**Purpura**	11 (78.56)	1 (7.1)
**Arthralgias**	13 (92.96)	4 (28.57)
**Neuropathic symptoms**	7 (50)	3 (21.43)
**Renal involvment**	2 (14.28)	1 (7.1)
**Skin ulcers**	1 (7.1)	0
**Sicca syndrome**	7 (50)	5 (37.71)
***Biohumoral***		
**Cryocrit**	3.36 (1–12)	1.46 (0–6)
**Rheumatoid factor**	12 (85.71)	10 (71.43)
**Reduced C4**	9 (64.3)	5 (37.71)

The diagnosis of liver disease was performed according to standard (histologic and/or clinical and ultrasound) criteria [25,26]. A diagnosis of liver cirrhosis, according to histological and/or clinical and ultrasound data, was made in 5 patients.

The nature of the study was explained to patients, who provided written informed consent before the beginning of the study, in accordance with the principles of the Declaration of Helsinki (revision of Edinburgh, 2000), and the study was approved by the University of Florence Ethics Committee.

The patients were prospectively enrolled according to the following inclusion criteria: presence of detectable HCV-RNA; eligibility for RTX treatment; no antiviral or immunosuppressive therapies (at least in the 6 months prior to enrolment); abnormal levels of liver enzymes; exclusion of Child-B/C cirrhosis; absence of hepatocellular carcinoma and acute viral hepatitis (<6 months); no co-infection with hepatitis B virus or human immunodeficiency virus, metabolic liver disease, vascular disease of the liver and biliary tract disorders; absence of an average daily alcohol consumption >50 g/day or use of hepatotoxic drugs; absence of clinical conditions potentially affecting TE, i.e. cardiac failure, and absence of physiological states contraindicated for this technique (i.e. pregnancy).

The administration of RTX was done according to the following regimen: intravenous infusion of 1 g at day 0 and at day 15. Before infusion, the patients received a pre-medication with acetaminophen, dexchlorpheniramine maleate and allopurinol. The concomitant use of steroids in high doses would be allowed only in case of hypersensitivity.

### Hepato-virological evaluation

The determination of HCV RNA in serum samples were performed by HCV-RNA Amplicor Monitor quantitative assay (Roche Diagnostics, Basel, Switzerland; limit of detection: 15 IU/ml), and the HCV RNA genotyping by INNO-LiPA HCV II, Immunogenetics, Gent, Belgium, according to the manufacturer instructions.

The degree of liver function was evaluated through biohumoral liver function tests (Alanine transaminase – ALT, Albumin, Alkaline phosphatase, Aspartate transaminase – AST, Bilirubin, Gamma-glutamyl transpeptidase, Lactic dehydrogenase, prothrombin time) and using the Child-Pugh score, as previously shown [27].

Liver biopsy was performed in 3 patients in order to confirm the fibrosis stage of liver disease. The patients underwent a measurement of liver stiffness by TE and ultrasound-guided percutaneous liver biopsy on the same day. Liver biopsy was performed on the right lobe of the liver, with a 16-gauge semiautomatic modified Menghini needle system (BIOMOL; Hospital Service, Aprilia, Italy) under local anaesthesia. Liver specimens were formalin-fixed and paraffin-embedded for histological evaluation. Sections of liver tissue were stained with hematoxylin and eosin and sirius red, and examined by an experienced pathologist unaware of the results of liver stiffness. All liver specimens had a length > 25 mm and included at least 11 complete portal tracts, reflecting adequate standards [28]. Fibrosis (F) was staged on a five-point scale according to METAVIR. Necro-inflammatory activity (A) was also graded on a four-point scale according to the method proposed by the Metavir Cooperative Study Group [25].

The patients were evaluated by TE (FibroScan, Echosens, Paris-France), according to the manufacturer instructions, in the right intercostal spaces, before treatment with RTX, 15 days after the first infusion, at month 1, 3 and 6 after the end of therapy.

The median values of ten successful acquisitions, expressed in kilopascal (kPa), were considered representative of the liver stiffness. Only procedures with 10 successful acquisitions, with a success rate of at least 60%, and an interquartile range (IQR) lower than 30% of the median value were considered reliable.

### IL28B genotyping

IL28B genotyping was performed on DNA extracted by whole blood samples, using a specific custom TaqMan SNP-Genotyping Assay (Single Nucleotide Polymorphism (SNP): rs12979860; Applied Biosystem, Foster City, CA, USA) based on allele-specific dual-labelled probes on a Rotor Gene 6000 (Corbett Research, Sidney, Australia) [29].

### CD19+ B cell count assessment

The levels of B cells were evaluated through CD19+ cells count, at baseline, at month 1, 3 and 6 after RTX treatment, according to other evaluated parameters. The test was performed using a routine method by a Coulter^®^ Lh 700 Series Hematology Analyzer (Beckman Coulter).

### Statistical analysis

All results were expressed as mean ± standard deviation. The numerical comparison of continuous data was performed using the Wilcoxon signed-ranks test applied to two-sample, matched pairs. Statistical significance was set at a value of p < 0.05. Statistical analysis were obtained using statistical software Stata 11, (College Station, TX, USA) and SPSS v18 (SPSS Inc., Chicago, IL, USA ).

## Results

### Hepato-virological evaluation

The main parameters of the study population at baseline and at month 3 after treatment are summarized in Table 2.

All patients scored anti-HCV and HCV RNA positive. The mean viral count doubled at 3 months. Eleven patients were infected by HCV genotype 1, 2 patients by genotype 2a/2c and the remaining one by genotype 3a. Concerning the determination IL28B genotypes, the C/C allele was determined in 3 patients (27.27%), while the other patients scored positive for the C/T or T/T alleles.

A liver biopsy was performed in 3 patients before treatment. In these patients stiffness values (7.4, 11.80, and 17.50 kPa) reflected high necro-inflammatory activity in 2 cases (A2 F0, A2 F0, METAVIR score), and a cirrhosis in the remaining one.

### Liver stiffness and CD19+ levels before, during and after rituximab treatment

In the study population, the mean stiffness values before treatment were 20.44 ±17.29 kPa and the mean CD19+ levels 124.2 ± 56.15x10^6^ L (Table 2).

**Figure 1:**
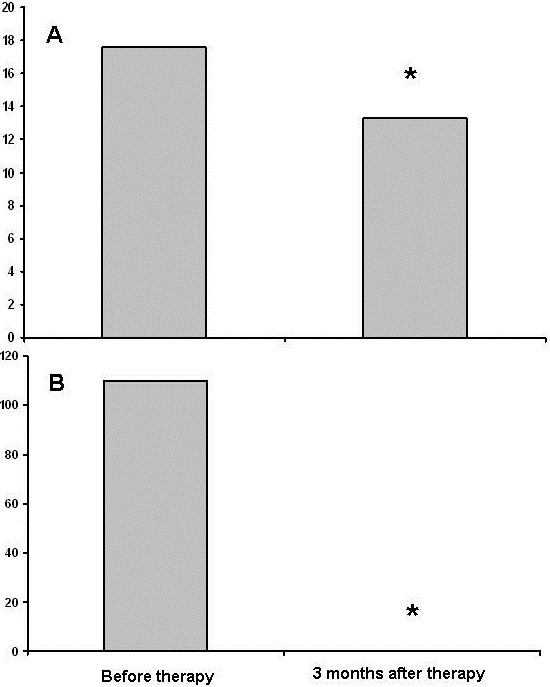
**Changes of liver stiffness values and CD19+ cell levels at baseline and at three months after the end of treatment in a singol patient.** Liver stiffness values (17.6 kPa before treatment vs 13.3 at month 3 after treatment) **(panel A)** and CD19+ levels (110 × 10^6^ L before treatment vs 0 × 10^6^ L at month 3 after treatment) **(panel B)**.

**Figure 2:**
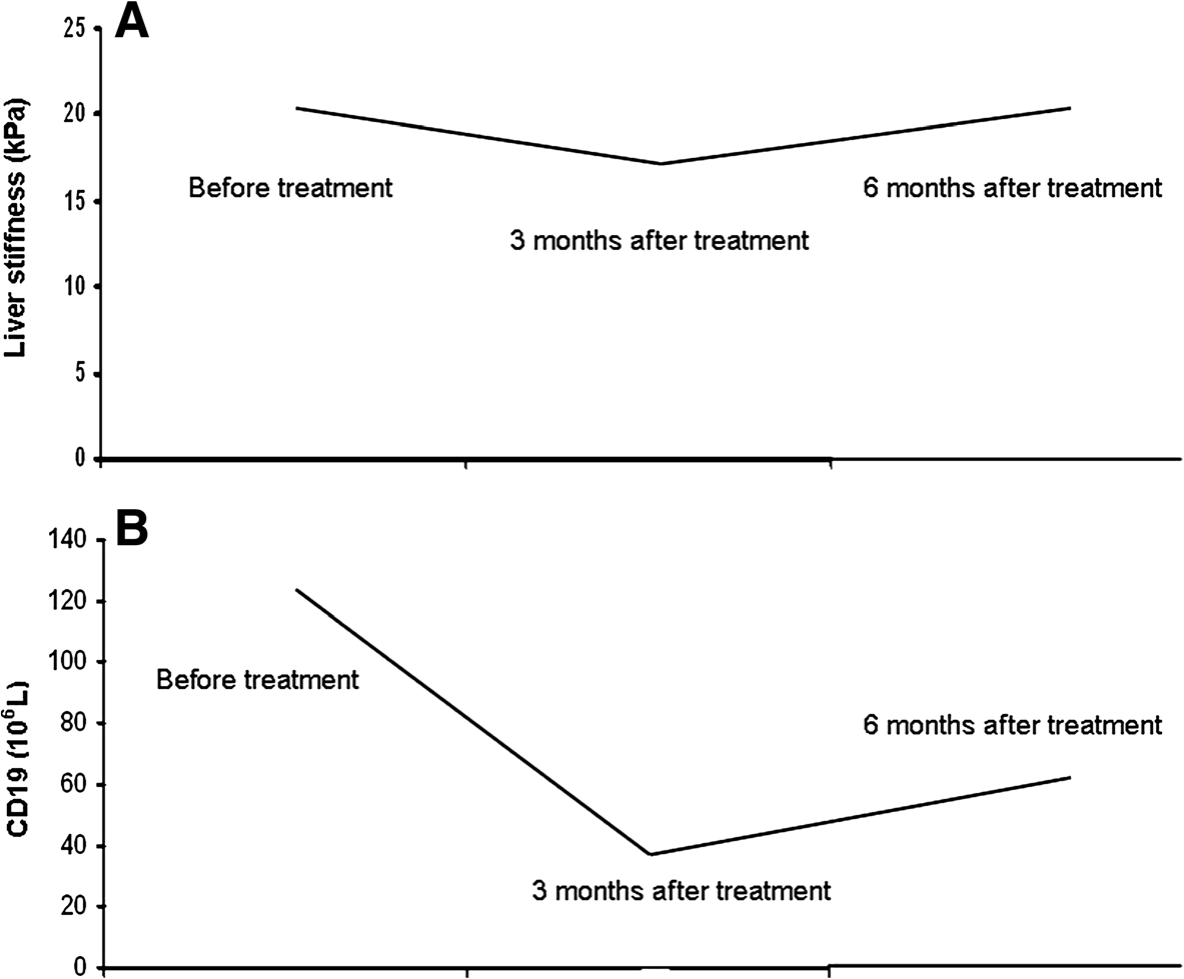
**Changes of liver stiffness values and CD19+ cell levels at baseline, at three months and at six months after the end of treatment.** Liver stiffness values **(panel A)** and CD19+ levels **(panel B)** were significantly different from those observed before treatment.

The patterns of liver stiffness during the study underwent changes that appeared related to the CD19+ levels (Table 2). Briefly: the stiffness values gradually decreased after therapy and were significantly lower three months after the end of treatment (p = 0.03). This was paralleled by the reduction of CD19+ levels (B-cell depletion) (p = 0.04), (Figures 1 and 2). Six months after treatment the liver stiffness came back to the baseline levels (p = 0.79) and the mean values of CD19+ gradually increased, even if they maintained a significant difference from those observed at baseline (p = 0.04) (Figure 2). Also principal mixed cryoglobulinemia manifestations (clinical and biohumoral) improved at months 3 after therapy (Table 1).

**Table 2:** Principal demographic and hepato-virological data of the 14 HCV-positive patients with mixed cryoglobulinemia before and 3 months after treatment with rituximab

**Parameter**	**Baseline**	**3 months**	**Normal range**	**P value**
Age (years)	60.43 ± 43		**Normal range**	
Gender (M%)	28.5			
BMI	26.24 ± 1.74		18–25	
Platelets (×10^9^ /L)	145.50 ± 71.22	163.4 ± 19.89	140–440	**p = 0.03**
RBC (×10^12^ /L)	4255 ± 438	4369 ± 723	4200–5400	p = 0.11
WBC (×10^9^ /L)	4684 ± 1374	4829 ± 1898	4–10	p = 0.86
ALT (U/L)	64.09 ± 39.66	65.63 ± 35.92	5–40	p = 0.86
AST (U/L)	69.54 ± 56.60	74.81 ± 40.78	5–40	p = 0.08
GGT (U/L)	66.09 ± 94.47	47.09 ± 30.03	10–40	p = 0.38
Creatinine (mg/dL)	0.70 ± 0.19	0.65 ± 0.15	0.6–1.5	p = 0.40
Glucose (mg/dL)	92.66 ± 11.58	92.33 ± 6.12	65–110	p = 0.32
Albumine (g/dL)	3.82 ± 0.35	4.05 ± 0.41	3.5–5.0	**p = 0.02**
Bilirubin (mg/dL)	0.88 ± 0.55	1.53 ± 1.99	0,3–1	p = 0.13
HCV-RNA (UI/L)	719798 ± 677364	1469311 ± 1576236	Undetectable	p = 0.07
Genotype 1	11			
Genotype 2a/2c	2			
Genotype 3	1			
CC polymorfism of IL-28-β	27.27%			
Mean stiffness (kPa)	20.44 ± 17.29	17.00 ±16.05		**p = 0.03**
CD 19+ (10^6^ L)	124.2 ± 56.15	38 ± 16.59		**p = 0.04**

*Abbreviations: BMI* Body mass index, *RBC* Red blood cells, *WBC* White blood cells, *ALT* Alanine transaminase, *AST* Aspartate aminotransferase, *GGT* Gamma glutamyl-transpeptidase. Statistically significant p values are labeled with bold text.

## Discussion

In the present study, for the first time, hepatic elastography was used to evaluate the liver stiffness values in HCV-related MC patients undergoing RTX therapy and the liver stiffness modifications were shown to be related to the CD19+ depletion. In previous studies [16,17,30-33] TE was shown to be an effective technique on one side for diagnosing severe fibrosis or cirrhosis (F3 or F4 according to METAVIR scoring system [25]) and, on the other, for excluding a significant fibrosis, strongly suggesting that it may be a “diagnostic discriminator” to establish clinical priorities and reduce the number of liver biopsies. Moreover, TE was demonstrated to be characterized by high intra- and inter-observer repeatability [34,35]. In recent years, some methods such us serum markers of liver fibrosis, TE and magnetic resonance (MR) have been proposed as non-invasive, easily reproducible and acceptable by patients also for assessing progression/regression of liver fibrosis. However, only the morphologic alterations associated with cirrhosis are well visualized by ultrasound or MR imaging. These techniques do not visualize the precirrhotic stage of liver fibrosis and the early cirrhosis. In these conditions, the liver parenchyma appear normal in MR imaging, but the administration of contrast agents improves the visibility of fibrosis. In particular, double contrast-enhanced MR imaging causes high image contrast between the low-signal-intensity fibrotic reticulations. The main limitation of this technique is the high cost and the inconvenience related with the use of two contrasts [36].

Recently, some studies [18,37-41] evaluated the use of TE for the assessment of short- and long-term longitudinal changes in liver stiffness during and after antiviral treatment in patients with chronic HCV infection. The results of these studies validated these techniques also in a longitudinal evaluation of liver stiffness values for monitoring treatment response and in clinical management of HCV chronic liver disease. However, no data exist concerning HCV MC patients treated with RTX. In the present study, the liver stiffness of patients with chronic HCV infection and MC undergoing RTX treatment, decreased concomitantly with CD19+ cell levels, particularly at month 3 after the end of therapy, suggesting the pivotal role played by the degree of liver infiltrates and subsequent liver necro-inflammation. This is in agreement with previous studies [16,17] showing that liver stiffness is influenced by necro-inflammatory activity. The important role played by B cells in both humoral and cellular immune response has been recently clarified [42,43]. Some studies [44,45] showed that CD19 + CD24hiCD27+ B cells regulate IFNγ production by CD4+ T cells via IL-10-dependent pathways and TNF-α production via IL-10 indipendent pathways. Antonelli et al. [46] in HCV-related MC showed significantly higher mean CXCL10 serum levels and TNF-alpha than HCV positive patients or controls. CXCL10 is secreted by the hepatocytes in inflammatory areas, and it recruits T cells to the hepatic lesions in chronic HCV infection [47]. Several studies [48-51] assessed the efficacy of RTX in patients with systemic sclerosis, because B cell play a key role in skin fibrogenesis [50,52]. The platelet-derived growth factor (PDGF) stimulates fibroblast proliferation, with subsequent collagen production. The study of Daoussis et al. [51] in eight systemic sclerosis patients demonstrated significant decrease of fibrosis in all six patients that showed histologic improvement after six months of RTX treatment. In these patients, the expression of PDGF receptors significantly decreased following RTX administration. This also suggests a potential role of RTX in a block of liver fibrosis progression during treatment.

In the study by Petrarca et al. [15], after RTX treatment, Resovist-enhanced magnetic resonance imaging was used to quantify the involvement of Kupffer cells in the clearance of large circulating immune complexes in MC. The authors showed that the iron signals were less than in healthy liver, but no evidence of significant modifications after RTX treatment were observed, suggesting that the improvement of cirrhotic syndrome was not consequence of an improved function of the reticuloendothelial system, but probably of the B cell depletion [15]. The results of the present study further support such an hypothesis.

Furthermore, concerning the potential risk of RTX treatment of patients with chronic viral infection, this study may confirm the safety of RTX in HCV patients with chronic liver disease. In fact, clinical/biohumoral parameters, including platelets, and albumine, in addition to liver stiffness, generally improved during therapy, thus confirming previous data [13,15,42].

Rituximab led to a response rate of 25% in B-cell chronic lymphocytic leukemia (B-CLL) patients [53]. In these patients it would be interesting to study the correlation between B cell expansion and liver stiffness in order to establish the effect of the drug on the liver.

In conclusion, this study for the first time showed that RTX-treatment in HCV MC and chronic liver damage induces a reduction of liver stiffness that is strictly associated with the B-cell depletion. This agrees with a previous observation [15] showing that RTX represents a safe and effective treatment of HCV-related MC also in patients with severe liver disease. Further studies will be usefull to better clarify the role played by B cells not only in the pathogenesis of HCV-related MC, but also of liver damage.

## Abbreviations

HCV: Hepatitis C virus; MC: Mixed cryoglobulinemia; MCS: Mixed cryoglobulinemia syndrome; NHL: Non-Hodgkin lymphoma; Peg-IFN: Pegylated interferon; RBV: Ribavirin; RTX: Rituximab; TE: Transient elastography; F: Fibrosis; A: Necro-inflammatory activity; MR: Magnetic resonance.

## Competing interest

The authors declare that they have no competing interest.

## Authors’ contributions

CS performed the research, collected and analysed the data, designed the research study and wrote the paper, ET performed the research and contributed to the drafting of the manuscript, UA, TU, AP performed the research and collected data, LG and GL revised it critically, ALZ contributed to the design and the writing of the paper and revised it critically. All authors read and approved the final manuscript.
